# Real-world effectiveness of early insulin therapy on the incidence of cardiovascular events in newly diagnosed type 2 diabetes

**DOI:** 10.1038/s41392-024-01854-9

**Published:** 2024-06-06

**Authors:** Sihui Luo, Xueying Zheng, Wei Bao, Sheng Nie, Yu Ding, Tong Yue, Yilun Zhou, Ying Hu, Hua Li, Qiongqiong Yang, Qijun Wan, Bicheng Liu, Hong Xu, Guisen Li, Gang Xu, Chunbo Chen, Huafeng Liu, Yongjun Shi, Yan Zha, Yaozhong Kong, Guobin Su, Ying Tang, Mengchun Gong, Linong Ji, Fan Fan Hou, Jianping Weng

**Affiliations:** 1 Department of Endocrinology, First Affiliated Hospital of USTC, Division of Life Sciences and Medicine, University of Science and Technology of China, Heifei, China; 2 Institute of Public Health Sciences, Division of Life Sciences and Medicine, University of Science and Technology of China, Hefei, China; 3 Division of Nephrology, National Clinical Research Center for Kidney Disease, State Key Laboratory of Organ Failure Research, Nanfang Hospital, Southern Medical University, Guangzhou, China; 4 Department of Nephrology, Beijing Tiantan Hospital, Capital Medical University, Beijing, China; 5 The Second Affiliated Hospital of Zhejiang University School of Medicine, Hangzhou, China; 6 Sir Run Run Shaw Hospital, Zhejiang University School of Medicine, Hangzhou, China; 7 Department of Nephrology, Sun Yat-Sen Memorial Hospital, Sun Yat-Sen University, Guangzhou, China; 8 The Second People’s Hospital of Shenzhen, Shenzhen University, Shenzhen, China; 9 Institute of Nephrology, Zhongda Hospital, Southeast University School of Medicine, Nanjing, China; 10 Children’s Hospital of Fudan University, Shanghai, China; 11 Renal Department and Institute of Nephrology, Sichuan Provincial People’s Hospital, School of Medicine, University of Electronic Science and Technology of China, Sichuan Clinical Research Center for Kidney Diseases, Chengdu, China; 12 Division of Nephrology, Tongji Hospital, Tongji Medical College, Huazhong University of Science and Technology, Wuhan, China; 13 Department of Critical Care Medicine, Maoming People’s Hospital, Maoming, China; 14 Key Laboratory of Prevention and Management of Chronic Kidney Disease of Zhanjiang City, Institute of Nephrology, Affiliated Hospital of Guangdong Medical University, Zhanjiang, China; 15 Huizhou Central People’s Hospital, Sun Yat-Sen University, Huizhou, China; 16 Guizhou Provincial People’s Hospital, Guizhou University, Guiyang, China; 17 Department of Nephrology, the First People’s Hospital of Foshan, Foshan, China; 18 Department of Nephrology, Guangdong Provincial Hospital of Chinese Medicine, the Second Affiliated Hospital, the Second Clinical College, Guangzhou University of Chinese Medicine, Guangzhou, China; 19 The Third Affiliated Hospital of Southern Medical University, Guangzhou, China; 20 Institute of Health Management, Southern Medical University, Guangzhou, China; 21 Department of Endocrinology and Metabolism, Peking University People’s Hospital, Beijing, China

**Keywords:** Endocrine system and metabolic diseases, Cardiovascular diseases

## Abstract

Early insulin therapy is capable to achieve glycemic control and restore β-cell function in newly diagnosed type 2 diabetes (T2D), but its effect on cardiovascular outcomes in these patients remains unclear. In this nationwide real-world study, we analyzed electronic health record data from 19 medical centers across China between 1 January 2000, and 26 May 2022. We included 5424 eligible patients (mean age 56 years, 2176 women/3248 men) who were diagnosed T2D within six months and did not have prior cardiovascular disease. Multivariable Cox regression models were used to estimate the associations of early insulin therapy (defined as the first-line therapy for at least two weeks in newly diagnosed T2D patients) with the incidence of major cardiovascular events including coronary heart disease (CHD), stroke, and hospitalization for heart failure (HF). During 17,158 persons years of observation, we documented 834 incident CHD cases, 719 stroke cases, and 230 hospitalized cases for HF. Newly diagnosed T2D patients who received early insulin therapy, compared with those who did not receive such treatment, had 31% lower risk of incident stroke, and 28% lower risk of hospitalization for HF. No significant difference in the risk of CHD was observed. We found similar results when repeating the aforesaid analysis in a propensity-score matched population of 4578 patients and with inverse probability of treatment weighting models. These findings suggest that early insulin therapy in newly diagnosed T2D may have cardiovascular benefits by reducing the risk of incident stroke and hospitalization for HF.

## Introduction

Type 2 diabetes (T2D) is an known risk factor for cardiovascular morbidity and mortality.^1^ In the past decade, results from cardiovascular outcome trials reveal that different glucose-lowering regimens have varying cardiovascular effects; some agents, such as glyburide and glipizide, increase the risk of cardiovascular and cerebrovascular events,^2^ while other agents, notably sodium glucose cotransporter 2 inhibitors (SGLT2is) and glucagon-like peptide 1 receptor agonists (GLP-1 RAs), demonstrate beneficial effect in reducing major cardiovascular events.^1^ As such, clinical guidelines emphasize the importance of reducing cardiovascular events, beyond lowering blood glucose level per se when choosing pharmacological agents for the management of T2D.^3^

Among the many available pharmaceutical options to treat T2D, insulin remains a highly potent regimen for glycemic control since its discovery.^1,3^ How insulin use affects cardiovascular outcomes in patients with T2D have gained immerse attention.^4–7^ Traditionally, insulin is used as the last resort among pharmaceutical options for T2D patients, when other treatments fail to control hyperglycemia.^3^ In patients who already had a longer duration of T2D and more chronic exposure to hyperglycemia, reports suggest the effects of such “late” insulin treatment regarding cardiovascular outcomes.^5,8,9^ On the other hand, a number of pilot studies suggest that early use of insulin therapy in newly diagnosed T2D patients achieves euglycemia and potentially repairs damaged β-cell function.^10–12^ These findings raised the question on the timing of initiation insulin therapy. Subsequent series of clinical and laboratory studies confirms that early insulin therapy not only succeeds in glycemic control, induces glycemic remission,^13–16^ but also restores β-cell function^15–17^ as well as relieves insulin resistance.^18^ Base on these evidences, increasing effort has been put into the investigation of the underlying mechanism of the benefit from early insulin therapy, defined as using insulin as the first-line hypoglycemic treatment for two weeks or longer in newly diagnosed T2D patients.^19^ The findings includes that such early insulin therapy improves biomarkers related to low-grade inflammation^20^ and endothelial function,^21^ which are known markers for cardiovascular risk, in newly diagnosed T2D patients. However, the effects of early insulin therapy in newly diagnosed T2D patients remains uncovered. Since 2010, early insulin therapy has been recommended in China as a first-line treatment option for newly diagnosed T2D patients, particularly when their glycated hemoglobin (HbA1c) level is greater than 9% (75 mmol/mol).^22^ It is imperative to take this unique opportunity to study the potential long-term cardiovascular effects of such early insulin therapy in T2D patients.

In this study, we aimed to evaluate the real-world effectiveness of early insulin therapy on the risk of major cardiovascular events including coronary heart disease (CHD), stroke, and hospitalization due to heart failure (HF) in newly diagnosed T2D, based on a nationwide collaborative network in China.

## Research design and methods

### Data source

Our data source was a nationwide collaborative network collecting electronic health records of hospitalization and out-patient clinic visits from 19 general medical centers of tertiary level across China between January 1, 2000, to May 26, 2022^23^ and has been described in previous publications.^24^ Briefly, in this collaborative network of hospitals, the de-identified raw data from each center were collected, pooled, and then standardized by trained healthcare workers and professional engineers from Digital Health China Technologies Co., LTD (Beijing, China). All the participating centers are requested to have their laboratories pass the annual External Quality Assessment of the Chinese National Center for Clinical Laboratories. Further quality control protocols have been executed to ensure data quality. Currently, data cleaning has been completed for nine modules of data, including personal information; major vital signs of each visit; visit details; diagnosis information coded with the 10th revision of the International Statistical Classification of Diseases and Related Health Problems (ICD-10 codes); surgical procedure information; drug prescriptions classified with anatomical therapeutic chemical (ATC) codes; other prescription details; laboratory results; and endpoint events of interest. All data are securely stored and available for access at the National Clinical Research Center for Kidney Disease in Guangzhou, China. By the time we accessed the dataset in July 2022, there are records of 7,084,405 participants available for analysis.

The Medical Ethics Committee of Nanfang Hospital, Southern Medical University has given approval to the protocol of the present study (approval number: NFEC-2019-213) and the individual informed consent were waived.

### Study population

We included newly diagnosed T2D participants who were diagnosed as T2D for no more than six months at the baseline visit. The baseline visit was defined as the record in which a participant was first diagnosed as T2D, or the first record of a participant in the database who was already diagnosed as T2D with a physician-documented diabetes duration no more than six months. The index day was defined as the discharge day if the baseline visit was hospital admission or the visit day if the baseline visit was to the outpatient clinic.

Patients diagnosed with T2D (ICD-10 code E11) for no more than six months at baseline met the inclusion criteria. We excluded those: (1) who had a diagnosis of type 1 diabetes or other types of diabetes (ICD-10 codes E10 or E13) in any record present in the database; (2) were pregnant at baseline; (3) with major medical illnesses including malignancy, organ transplant, end-stage renal or liver disease at baseline; (4) who were lost to follow-up after the index day; and (5) who had prior diagnosis of cardiovascular disease (CVD) at baseline, defined as having CHD (ICD 10 codes I20 – I25), HF (ICD-10 code I50), or cerebrovascular disease (ICD-10 codes I60 – I69).

### Exposure

The exposure was early insulin therapy at baseline, which was defined as using subcutaneous insulin therapy in patients within six months of diagnosed of T2D. The thereapy should have lasted for at least two weeks, with or without other antihyperglycemic drugs. The duration of insulin therapy is defined in align with previous trials^13–16^ of short-term intensive insulin therapy inducing T2D remission. Insulin therapy was identified using the ATC code A10, regardless of insulin type. Codes for other antihyperglycemic agents can be found in the Supplementary materials, Table S1.

### Outcome measures

The outcomes included the incidence of three cardiovascular outcomes: CHD (ICD-10 codes I20–I25), stroke (ischemic and hemorrhagic stroke, ICD-10 codes I60–I64), and hospitalization for HF (ICD-10 codes I50). Details of definition of these outcomes were described in the Supplementary materials, Table S2.

### Statistical analyses

Descriptive statistics were summarized for the study participants’ baseline characteristics. The *t* test (continuous variables, normal distribution), the Mann–Whitney *U* test (continuous variables, skewed distribution), and the Chi-square test (categorical variables) were used to compare T2D patients who received early insulin therapy versus those who did not. For the imputation of continuous data, multiple imputation by chained equations with predictive mean-matching techniques was applied.

Time-to-event distributions were summarized with Kaplan-Meier curves. We used Cox proportional hazards models to compare the risks of cardiovascular outcomes between study participants who received early insulin therapy and those who did not. We estimated the association between cardiovascular events of interest and early insulin therapy by calculating hazard ratios (HRs) and 95% confidence intervals (CIs). Follow-up time was computed from the index day to the date of cardiovascular events, death, or May 26 2022, whichever came first. The proportional hazard assumptions were examined using Schoenfeld residuals, and as a result, we censored the data at 140 months (11.7 years) since the index day for all the analyses to allow adherence to the proportional hazard assumptions. Models were adjusted for potentially confounding factors, including baseline demographic, comorbidity, and laboratory characteristics; ever use of statins, antiplatelet and antihypertension agents any time during follow-up; as well as baseline use of antihyperglycemic agents. These variables were ascertained from the structuralized electronic records. In particular, the use of statins, antiplatelet and antihypertension agents was identified from the prescription data using ATC codes shown in the Supplementary materials, Table S1.

We performed several prespecified sensitivity analyses to evaluated the robustness of our results. First, we performed the analyses for each of the outcomes in a propensity score matched (PSM) population, with 1:1 matching according to the patients’ age, gender, BMI, baseline HbA1c level, and baseline estimated glomerular filtration rate (eGFR) determined by the modification of diet in renal disease (MDRD) equation (revised version for the Chinese population), systolic blood pressure, serum high-density lipoprotein cholesterol (HDL-C), serum low-density lipoprotein cholesterol (LDL-C), and the baseline use of metformin, statin and antiplatelet agents.^25^ The two groups’ baseline characteristics in the PSM population were compared using the standardized mean difference (SMD), in order to assess the balance between groups. We consider the variables well matched when SMD < 0.1.^26^ Second, we examine the association using inverse probability of treatment weighting (IPTW) analysis. In the IPTW analysis, the inverse probability weighting weight for every participant was determined using the anticipated probabilities from the propensity-score model, and this weight was then applied in multivariable cox regression models. Third, to account for the potential influence of reverse causation, we repeated the analyses after excluding the outcome events occurring within 90 days and 180 days from the index day among the participants who received early insulin therapy and who did not. Also, to investigate whether the effect of early insulin therapy on CVD could be attributed to better glycemic control, we have further adjusted for the mean HbA1c levels six months after the treatment. To further rule out the potential confounding from cardiovascular protective effect exerted by GLP-1 RAs and SGLT2is, we have repeated the analysis excluding patients who had ever used GLP-1 RAs or SGLT2is in both groups.

In addition, we conducted stratified analyses by age (<60 years vs. ≥60 years), gender, body mass index (BMI, <24 vs. 24–27.9 vs. ≥28 kg/m^2^), diagnosis of hypertension at baseline, baseline HbA1c levels (>9% [75 mmol/mol] vs. ≤9% [75 mmol/mol]), baseline serum LDL-C level (≥2.6 mmol/L vs. <2.6 mmol/L), baseline use of metformin, ever use of statins, and ever use of antiplatelets.

*P* < 0.05 was considered statistically significant in all analyses. SAS 9.4 (SAS Institute Inc., Cary, NC) and R 4.0.2 (http://www.r-project.org) were used for all statistical analyses.

## Results

### Summary of the study population

**Fig. 1:**
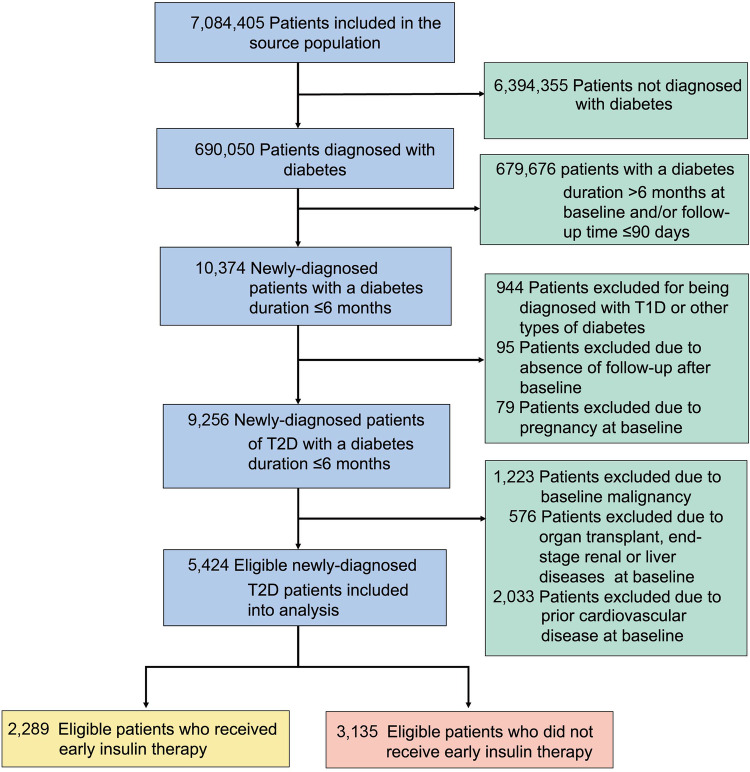
Flow chart for eligibility screening and inclusion of the study population. T2D type 2 diabetes, T1D type 1 diabetes

Of 7,084,405 participants in the network database when we performed the analysis, we identified 690,050 patients with diabetes. And through a sequential eligibility assessment, (Fig. 1) we finally included 5424 eligible patients with newly diagnosed T2D (within six months), who did not have prior CVD. Among them, we grouped these patients according to whether they received early insulin therapy. We then identified 2289 patients who received early insulin therapy and 3135 patients who did not (Fig. 1).

The participants’ baseline characteristics were summarized in Table 1. Compared with the patients who did not receive early insulin therapy, the patients who received such treatment had a higher level of HbA1c (mean [standard deviation, SD], 9.73 [3.02] vs. 8.44 [2.89], %) and fasting plasma glucose (median [interquartile range, IQR] 9.16 [6.09, 14.2] vs. 8.44 [5.95, 13.20], mmol/L), were slightly younger (mean [SD] 54.57[13.27] vs. 57.22[12.77], years), and had a higher proportion of concomitant use of metformin (49.1% vs 26.2%) and dipeptidyl peptidase-4 inhibitors (11.9% vs 3.48%) at baseline.

**Table 1:** Baseline characteristics of the included patients with newly diagnosed type 2 diabetes

	Early insulin therapy		*p* value
	No	Yes	
*N*	3135	2289	–
Age, mean (SD), years	57.22 (12.77)	54.57 (13.27)	<0.001
<40	269 (8.58)	319 (13.9)	–
40–59	1536 (49.0)	1148 (50.2)	–
≥60	1330 (42.4)	822 (35.9)	–
Gender – female, no. (%),	1326 (42.3)	850 (37.1)	<0.001
Body mass index, mean (SD), kg/m^2^	24.71 (10.84)	24.82 (11.03)	0.254
<24	1118 (35.7)	810 (35.4)	–
24–27.9	1517 (48.4)	1076 (47.0)	–
≥28	500 (15.9)	403 (17.6)	–
Comorbidities			
Atrial fibrillation	103 (3.29)	48 (2.10)	0.011
COPD and/or pulmonary vascular disease	99 (3.16)	31 (1.35)	<0.001
Hypertension	913 (29.1)	565 (24.7)	<0.001
SBP, mean (SD), mmHg	134.72 (36.62)	133.82 (46.50)	0.424
DBP, mean (SD), mmHg	82.17 (26.60)	82.37 (28.56)	0.782
HbA1c, mean (SD), no. (%)	8.44 (2.89)	9.73 (3.02)	<0.001
Fasting plasma glucose, median (IQR), mmol/L	8.44 (5.95–13.2)	9.16 (6.09–14.2)	0.001
Serum creatinine, median (IQR), μmol/L	72.9 (60.0–89.0)	70.0 (57.0–89.0)	<0.001
eGFR, median (IQR), ml/min/1.73 m^2^	91.0 (74.1–113)	97.9 (75.5–125)	<0.001
Serum total cholesterol, mean (SD), mmol/L	5.02 (1.58)	5.03 (1.80)	0.800
Serum HDL-C, mean (SD), mmol/L	1.15 (0.35)	1.08 (0.36)	<0.001
Serum LDL-C, mean (SD), mmol/L	3.02 (1.14)	2.89 (1.11)	<0.001
Serum triglyceride, mean (SD), mmol/L	2.16 (2.78)	2.42 (3.60)	0.002
Concomitant antihyperglycemic drugs, no. (%)			
Metformin	822 (26.2)	1125 (49.1)	<0.001
Sulfonylureas	373 (11.9)	385 (16.8)	<0.001
α-glucosidase inhibitors	929 (29.6)	885 (38.7)	<0.001
DPP4is	109 (3.48)	272 (11.9)	<0.001
GLP-1 RAs	12 (0.38)	28 (1.22)	0.001
SGLT2is	16 (0.51)	77 (3.36)	<0.001
TZDs	125 (3.99)	76 (3.32)	0.226
Non-SU insulin secretagogues	373 (11.9)	302 (13.2)	0.166
ACEIs/ARBs, no. (%)	1155 (36.8)	823 (36.0)	0.521
CCBs, no. (%)	1173 (37.4)	820 (35.8)	0.241
β-blockers, no. (%)	817 (26.1)	510 (22.3)	0.002
Diuretics, no. (%)	860 (27.4)	616 (26.9)	0.693
Statins, no. (%)	1685 (53.7)	1212 (52.9)	0.579
Antiplatelet drugs, no. (%)	1271 (40.5)	819 (35.8)	<0.001
Duration of early insulin therapy, medium (IQR), days	NA	35.0 (17.8–81.7)	NA

Data are presented as number (%), mean (standard deviation, SD), or median (interquartile range, IQR)

*COPD chronic obstructive pulmoriary disease,* SBP systolic blood pressure, *DBP* diastolic blood pressure; *HbA1c* hemoglobin A1c, *eGFR* estimated glomerular filtration rate, *HDL-C* high-density lipoprotein cholesterol, *LDL-C* low-density lipoprotein cholesterol, *DPP4i* dipeptidyl peptidase 4 inhibitor, *GLP-1 RA* glucagon-like peptide-1 receptor agonist, *SGLT2i* sodium-glucose cotransporter-2 inhibitor, *TZD* thiazolidinedione, *Non-SU insulin secretagogue* non-sulfonylurea insulin secretagogue, *CCB* calcium channel blocker, *ACEI/ARB* angiotensin-converting enzyme inhibitor/angiotensin II receptor blocker

### Association of early insulin therapy with cardiovascular outcomes

**Fig. 2:**
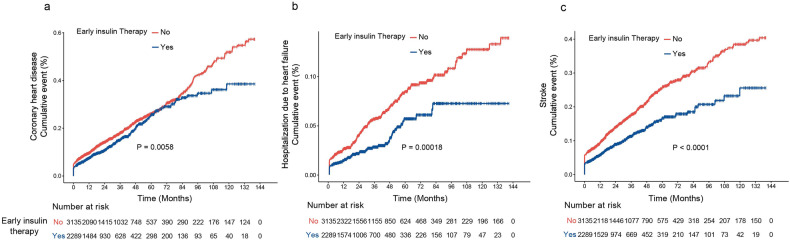
Time-to-event distribution for (**a**) coronary heart disease (CHD), (**b**) hospitalization for heart failure (HF), and (**c**) stroke in the study population

During 17,158 persons years of observation (mean duration of observation, 39.6 months), we documented 834 incident CHD cases, 719 stroke cases, and 230 hospitalized cases for HF. Individuals who received early insulin therapy were less likely to develop CHD (12.8% vs. 17.3%), hospitalization for HF (2.75% vs. 5.32%), and stroke (9.4% vs. 16.1%) compared with those who did not receive early insulin therapy. Time-to-event distribution was displayed in Fig. 2a–c.

In the multivariable Cox regression models, early insulin therapy among newly diagnosed T2D patients was associated with a 31% lower risk of incident stroke (hazard ratio [HR] 0.69, 95% confidence interval [CI] 0.58–0.81) and a 28% lower risk of hospitalization for HF (HR 0.72, 95% CI 0.53–0.99), and had a null association with CHD (HR 1.08, 95% CI 0.92–1.28), after adjusted for potentially confounding factors, including the centers where the patients were treated, baseline demographic, comorbidity, and laboratory characteristics; ever use of statins, antiplatelet and antihypertension agents any time during follow-up; as well as baseline use of antihyperglycemic agents (Table 2).

**Table 2:** Hazard ratios for the association between early insulin therapy users versus non-early insulin therapy users and the risk of cardiovascular outcomes

	Early insulin therapy		*p* value
	No	Yes	
No. of patients	3135	2289	
Outcomes			
Coronary heart disease			
No. of events	542	292	
Follow-up time (persons years)	8639	5394	
Hazard ratio (95% CI)^a^			
Model 1	1.00 (Reference)	0.91 (0.79–1.06)	0.218
Model 2	1.00 (Reference)	1.00 (0.86–1.15)	0.958
Model 3	1.00 (Reference)	1.07 (0.92–1.25)	0.398
Model 4	1.00 (Reference)	1.08 (0.92–1.28)	0.367
Hospitalization for heart failure			
No. of events	167	63	
Follow-up time (persons years)	9652	5857	
Hazard ratio (95% CI)^a^			
Model 1	1.00 (Reference)	0.66 (0.49–0.89)	<0.001
Model 2	1.00 (Reference)	0.68 (0.50–0.92)	<0.001
Model 3	1.00 (Reference)	0.67 (0.49–0.91)	<0.001
Model 4	1.00 (Reference)	0.72 (0.53–0.99)	<0.001
Stroke			
No. of events	504	215	
Follow-up time (persons years)	9008	5626	
Hazard ratio (95% CI)^a^			
Model 1	1.00 (Reference)	0.71(0.60–0.83)	<0.001
Model 2	1.00 (Reference)	0.68 (0.58–0.80)	<0.001
Model 3	1.00 (Reference)	0.68 (0.57–0.80)	<0.001
Model 4	1.00 (Reference)	0.69 (0.58–0.81)	<0.001

^a^Hazard ratios were estimated using the following models: Model 1, adjusted for gender; baseline age and body mass index; Model 2, Model 1 and further adjusted for baseline history of hypertension, history of chronic obstructive pulmonary disease(COPD) and/or pulmonary vascular diseases, history of atrial fibrillation history, systolic blood pressure, HbA1c, estimated glomerular filtration rate (eGFR), serum high-density lipoprotein cholesterol (HDL-C) and serum low-density lipoprotein cholesterol (LDL-C); Model 3, Model 2 and further adjusted for ever use of statin, antiplatelet drugs, diuretics, angiotensin converting enzyme inhibitors/ angiotensin II inhibitors(ACEI/ARBs), β-receptor blocker and calcium channel blockers(CCBs) during follow-up; and baseline use of metformin, sulfonylureas, α-glucosidase inhibitors, thiazolidinediones, dipeptidyl peptidase 4 inhibitors, glucagon-like peptide-1 receptor agonists and sodium-glucose cotransporter-2 inhibitors; Model 4, Model 3 and further adjusted for the centers where the patients were treated

*CI* confidence interval

### Sensitivity analyses

Sensitivity analyses showed the robustness of our findings. First, the propensity-score matching analysis matched for baseline age, gender, BMI, HbA1c and eGFR, systolic blood pressure, serum HDL-C, serum LDL-C, and the baseline use of metformin, statin and antiplatelet agents. The characteristics of the 1:1 propensity score-matched population were shown in the Supplementary materials, Table S3. The results of the multivariable-adjusted Cox regression models showed that, consistent with the main analysis results, early insulin therapy was associated with a 31% lower risk of stroke (HR 0.69, 95% CI 0.56–0.85) and a 36% lower risk of hospitalization for HF (HR 0.64, 95% CI 0.45–0.92), and not associated with the risk of CHD (HR 1.10, 95% CI 0.91–1.32). (Supplementary materials, Table S4). Second, in the inverse probability of treatment weighing (IPTW) models, early insulin therapy was also associated with a 31% lower risk of stroke (HR 0.69, 95% CI 0.58–0.82) and a 33% lower risk hospitalization for HF (HR 0.67, 95% CI 0.47–0.95). No difference was observed for the risk of CHD (HR 1.12, 95% CI 0.94–1.33). (Supplementary materials, Table S5) Third, similar results were found in the sensitivity analyses when we excluded the cardiovascular events of interest occurring within 90 days and 180 days since the index day to rule out the potential influence of reverse causation (Supplementary materials, Tables S6 and S7).

In addition, in the sensitivity analysis with further adjustment for the mean HbA1c level within 6 months after baseline, we found similar results: early insulin therapy was associated with a 31% lower incidence in hospitalization for HF (HR 0.69, 95% CI 0.55–0.86) and 19% lower incidence in stroke (HR 0.81, 95% CI 0.72–0.92; Table S8). Finally, similar findings were observed in another sensitivity analysis excluding the participants who had ever used GLP-1 RAs and SGLT2i during the whole follow-up (Table S9).

### Stratified analyses

Stratified analyses revealed that the lower risk associated with early insulin therapy on stroke appeared to be more profound among those who were with a BMI less than 28 kg/m^2^ (*P* for interaction 0.013), without hypertension at baseline (*P* for interaction 0.005), had a baseline HbA1c level ≤9% (75 mmol/mol) (*P* for interaction 0.010) and ever used statins to control blood lipid (*P* for interaction 0.044); and was attenuated among patients who ever took antiplatelet drugs (*P* for interaction 0.003), as shown in the Supplementary materials Fig. S1. For hospitalization for HF, shown in the Supplementary materials Fig. S2, the lower risk associated with early insulin therapy appeared to be more profound among patients who did not have a baseline diagnosis of hypertension (*P* for interaction 0.013) and was also attenuated among patients who ever took antiplatelet drugs. (*P* for interaction 0.048).

## Discussion

In this nationwide, multicenter, real-world study, we found that early insulin therapy was associated with a decreased incidence of cardiovascular events of interest, with a 31% lower risk of stroke and 28% lower risk of hospitalization for HF among patients with T2D-diagnosed <6 months.

To our knowledge, this study was the first to show a significant reduction of incident cardiovascular outcomes associated with early insulin therapy in patients with newly diagnosed T2D. Previous studies have shown that early insulin therapy in patients with newly diagnosed T2D may alter the clinical course of T2D by restoring β-cell function and promoting extended glycemic remission.^13–16^ In a previous multicenter, randomized trial to compare the effects of oral hypoglycemic agents and short-term intensive insulin therapy, we found that newly diagnosed T2D patients treated with early insulin therapy achieved greater target glycemic control using shorter time, having higher remission rates after one year, showing better acute insulin response and better β-cell function than those treated with oral hypoglycemic agents.^16^ Our findings in the present study also corroborated with the observation in the UK Prospective Diabetes Study (UKPDS)^27^ and the Veterans Affairs Diabetes Trial (VADT)^28^ that early intensive glycemic control in T2D reduces risk of cardiovascular outcomes. The UKPDS have shown that, at the ten-year follow-up after the trial, good glycemic control among patients with newly diagnosed T2D treated with insulin or sulfonylurea lead to a 9% (*P* = 0.04) of relative risk reduction (RRR) of diabetes-related composite endpoints (including fatal or non-fatal myocardial infarction, angina, heart failure, stroke, and other outcomes) and 15% RRR of myocardial infarction (*P* = 0.01). Recent post hoc analysis from the UKPDS cohort reveals that benefits of the initial antihyperglycemic treatment on CVD outcomes prevailed for many years after treatment cessation and the loss of between-group glycemic differences. In addition, subgroup analysis of the VADT indicates that T2D patients who had a time from diabetes diagnosis <15 years and received intensive glycemic control showed a reduced risk of cardiovascular events (HR range, 0.7–0.8).^29^ In contrast, a nationwide retrospective cohort study^4^ reported that using metformin added-on insulin was linked to an increased risk of all-cause mortality, compared with metformin plus glimepiride among T2D patients with an average diabetes duration of about 4.5 years who failed in glycemic control using metformin alone. We consider the discrepancy results from the longer duration of diabetes in this study compared with our study population. Also, the failure of metformin monotherapy in the patients in this study might suggest poorer glycemic control. In a subgroup analysis of the FREEDOM trial, patient with multiple coronary artery disease, patient treated insulin have higher risk of five-year event rate for MI, stroke, and death.^30^ We speculate that the difference in the findings can be attributed to the much longer duration of diabetes in the FREEDOM trial participants and likely their prior CVD history. A more recent investigation in a Korean population ascertaining 534 insulin-treated patients and 534 oral antidiabetic drugs-treated patients reveals that early initiation of insulin therapy was linked a decreased but not statistically significant risk of incident stroke (13 cases vs. 17 cases, HR 0.73, 95% CI 0.5–1.50) and ischemic heart disease (46 cases vs. 46 cases, HR 0.95, 95% CI 0.63–1.44) compared with the oral antidiabetic drugs treatment.^31^ The non-significant findings may be mainly due to the limited number of outcome cases identified (i.e., 13 cases vs. 17 cases for stroke and 46 cases vs. 46 cases for ischemic heart disease between groups). Based on the above discussion, we could reasonably assume that achieving early metabolic control via early insulin therapy may be the key regarding its cardiovascular protective effect.

The observed cardiovascular benefits related to early insulin therapy are biologically plausible. Mechanism studies have confirmed that β-cell function restoration via redifferentiation underlies the short-term intensive insulin treatment that results in diabetic remission: loss of β-cell mass in T2D was due to β-cell dedifferentiation, not apoptosis;^32^ and following insulin therapy lowers blood glucose, the dedifferentiated β-cells can later redifferentiate to mature β-cells that restores insulin-secreting function.^17^ Diabetes remission is accompanied by the relief of lipotoxicity and insulin resistance, which are both well known risk factors for CVD development.^33^ Furthermore, a recent study has shown that short-term, treat-to-strict-glycemic-target insulin therapy applied to newly diagnosed T2D patients downregulates the expression of pro-inflammatory cytokines including interleukin-6 receptor and intercellular adhesion molecule-1, which are biomarkers of endothelial dysfunction.^20^ Inflammation and endothelial dysfunction are key factors in the pathogenesis of atherosclerosis and cardiovascular disease. Insulin exerts a vasodilatory effect through endothelial nitrogen monoxide (NO) release in arteries, veins, and capillaries.^34,35^ Gao et al. first showed that insulin suppresses tumor necrosis factor α (TNF-α) production both locally and systemically in myocardial ischemia/reperfusion rat models. They found that insulin administration in vitro reduced the generation of TNF-α in cardiomyocytes generated by ischemia/reperfusion through the Akt-eNOS-NO signaling pathway.^36^ But the reason that the effect appeared to be more prominent on stroke and hospitalization for heart failure, but not on CHD, remains unclear and warrants further investigation.

In addition, the Outcome Reduction with an Initial Glargine Intervention (ORIGIN) Trial examined the use of basal insulin (insulin glargine) in patients with prediabetes or established T2D (mean diabetes duration of five years) compared with standard care and showed insulin glargine had a neutral effect on the risk of cardiovascular events including stroke and myocardial infarction (HR 1.02).^37^ The findings in the ORIGIN trial highlighted the safety of basal insulin use. The discrepancies of the effect of insulin on cardiovascular events between the ORIGIN trial and the present study may be due to the difference in diabetes duration and pre-existing CVD status at baseline of the study population. Also, among the participants in the present study, some patients receiving early insulin therapy may basically require insulin therapy. This typically occurs among patients who experience poor glycemic control with other treatments. Such patients are known to have higher risk of CVD. Still, we observed that early insulin therapy is linked to a lower risk of cardiovascular events, suggesting that the real correlation between early insulin use and cardiovascular outcomes may be stronger than the observed association in the present study. In summary, our study provides evidence that early insulin therapy as an initial treatment, not a late option, may yield substantial clinical benefits among type 2 diabetes patients.

A major strength of this study is that it was based on a nationwide collaborative network, which facilitates the generalization of the findings to a broader population. There are some limitations in this study. First, due to the non-randomization nature of this real-world study, the observed findings may be subject to confounders. However, our findings were not appreciably altered after we adjusted extensively to potential confounders, including comorbidities and concomitant medications such as statins, antiplatelets, β-blockers, diuretics, metformin, and the recently emerged GLP-1 RAs and SGLT2is. Moreover, sensitivity analyses with propensity score matching supported our findings’ robustness. Second, currently there are different insulin regimens available for use. More research is required to examine the association of different insulin regimens with CV events in newly diagnosed T2D. Third, even though we have taken a lot of potential confounders into account in this study, residual confounding by unmeasured or unknown confounders, such as smoking status, socioeconomic factors, and data on the change in β cell function, cannot be completely ruled out. Also, the sensitivity analysis showed that the benefit of early insulin therapy prevails after adjusting for mean HbA1c levels within six months after baseline. We assumed that preserved β cell function and thus better long-term glycemic control may contribute to such effect, which warrants further study.

In conclusion, early insulin therapy may have cardiovascular may benefits for patients with newly diagnosed T2D by reducing the risk of incident stroke and hospitalization for HF, supporting early insulin therapy as an initial option for newly diagnosed T2D.

### Supplementary information

Supplementary Materials

## Data Availability

No further data are available. Aggregated data is available on request to the corresponding authors.
