# Supplementary material for: Real-world effectiveness of early insulin therapy on the incidence of cardiovascular events in newly diagnosed type 2 diabetes
Source: Signal Transduct Target Ther. 2024 Jun 6;9:154. doi: 10.1038/s41392-024-01854-9 (PMC11156919; doi:10.1038/s41392-024-01854-9)
Supplement: Supplementary file 1 — Supplementary Materials [file 41392_2024_1854_MOESM1_ESM.docx]

Supplementary Materials for

Real-world effectiveness of early insulin therapy on the incidence of cardiovascular events in newly diagnosed type 2 diabetes—Cardiovascular Benefits of Early Insulin in New T2D

Sihui Luo M.D.^1*^, Xueying Zheng M.D.^1*^, Wei Bao MD. Ph.D.^1,2*^, Sheng Nie M.D.^3^, Yu Ding M.D.^1^, Tong Yue M.S.^1^, Yilun Zhou M.D.^4^, Ying Hu M.D.^5^, Hua Li M.D.^6^, Qiongqiong Yang Ph.D.^7^, Qijun Wan M.D.^8^, Bicheng Liu M.D.^9^, Hong Xu Ph.D.^10^, Guisen Li M.D.^11^, Gang Xu Ph.D.^12^, Chunbo Chen M.D.^13^, Huafeng Liu M.D.^14^, Yongjun Shi Ph.D.^15^, Yan Zha M.D.^16^, Yaozhong Kong M.D.^17^, Guobin Su M.D.^18^, Ying Tang M.D.^19^, Mengchun Gong, M.D.^20^, Linong Ji M.D.^21^, Fanfan Hou MD. Ph.D.^3^, Jianping Weng M.D. Ph.D.^1^

Correspondence to: wengjp@ustc.edu.cn

**This PDF file includes:**

Figures. S1 to S3

Tables S1 to S9

Figure. S1.

Stratified analysis of the incidence of stroke.


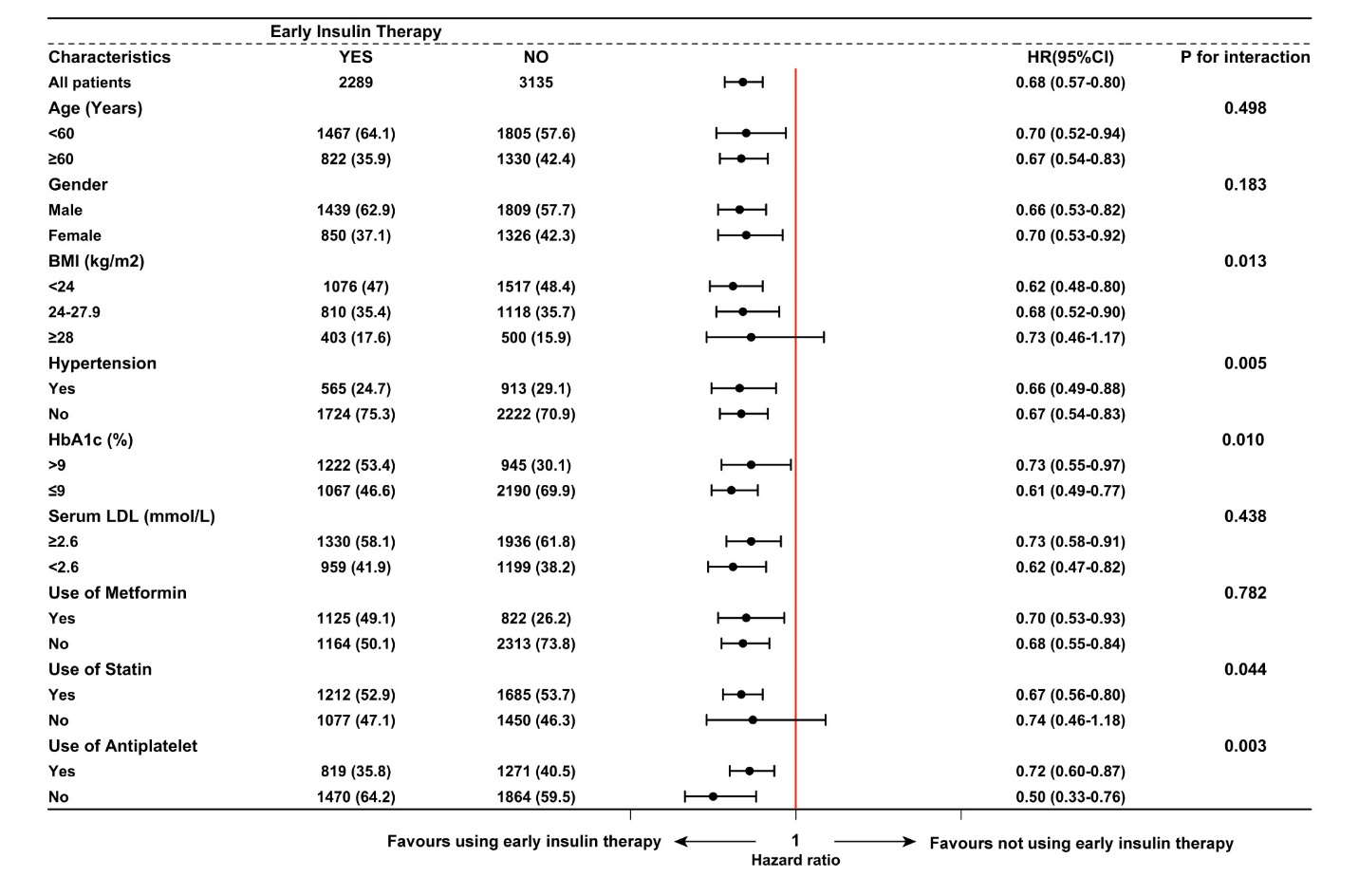


Abbreviations: HR, hazard ratio; CI, confidence interval; HbA1c, hemoglobin A1c; LDL, low density lipoprotein cholesterol.

Hazard ratios were adjusted for gender; baseline age, body mass index, history of hypertension, history of chronic obstructive pulmonary disease(COPD) and/or pulmonary vascular diseases, history of atrial fibrillation history, systolic blood pressure, estimated glomerular filtration rate (eGFR), high-density lipoprotein cholesterol (HDL-C), sulfonylureas, α-glucosidase inhibitors, thiazolidinediones, dipeptidyl peptidase 4 inhibitors, glucagon-like peptide-1 receptor agonists and sodium-glucose cotransporter-2 inhibitors; and ever use of statin, antiplatelet drugs, diuretics, angiotensin converting enzyme inhibitors/ angiotensin II inhibitors(ACEI/ARBs) , β-receptor blocker and calcium channel blockers(CCBs) during follow-up.

Figure. S2.

Stratified analysis of the incidence of hospitalization for heart failure.


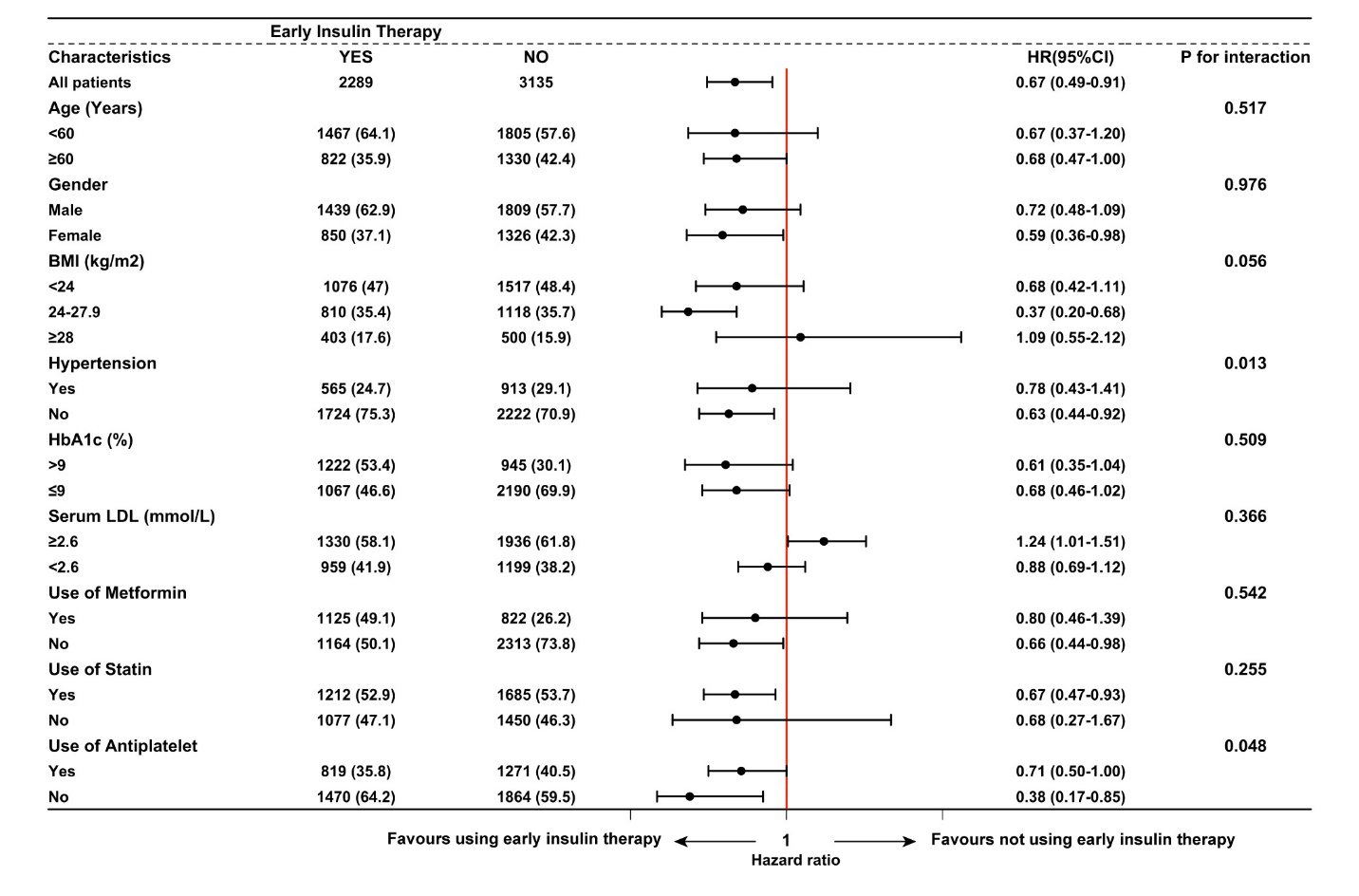


Abbreviations: HR, hazard ratio; CI, confidence interval; HbA1c, hemoglobin A1c; LDL, low density lipoprotein cholesterol.

Hazard ratios were adjusted for gender; baseline age, body mass index, history of hypertension, history of chronic obstructive pulmonary disease(COPD) and/or pulmonary vascular diseases, history of atrial fibrillation history, systolic blood pressure, estimated glomerular filtration rate (eGFR), high-density lipoprotein cholesterol (HDL-C), sulfonylureas, α-glucosidase inhibitors, thiazolidinediones, dipeptidyl peptidase 4 inhibitors, glucagon-like peptide-1 receptor agonists and sodium-glucose cotransporter-2 inhibitors; and ever use of statin, antiplatelet drugs, diuretics, angiotensin converting enzyme inhibitors/ angiotensin II inhibitors(ACEI/ARBs) , β-receptor blocker and calcium channel blockers(CCBs) during follow-up.

Figure. S3.

Stratified analysis of the incidence of coronary heart disease.


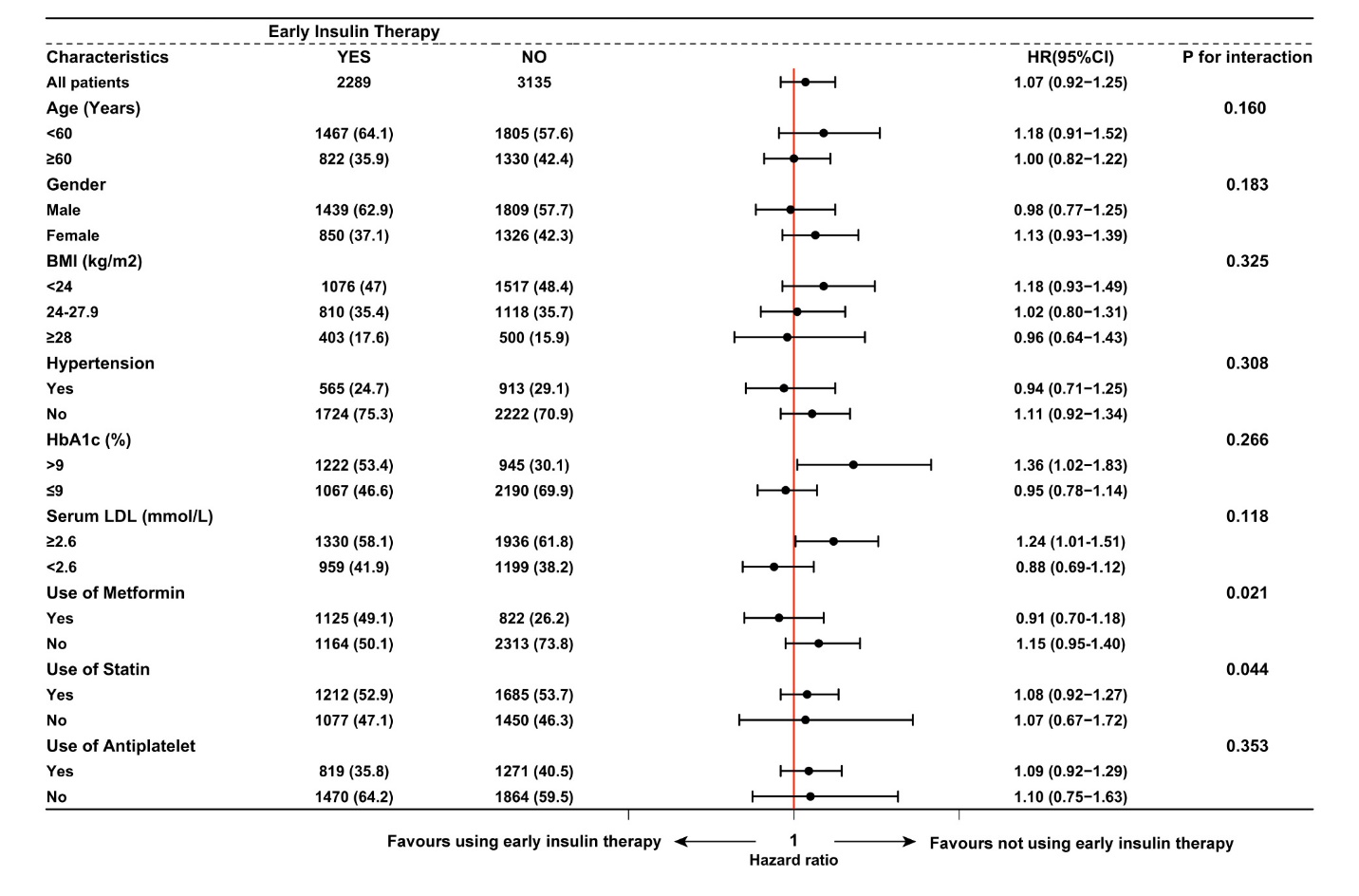


Abbreviations: HR, hazard ratio; CI, confidence interval; HbA1c, hemoglobin A1c; LDL, low density lipoprotein cholesterol.

Hazard ratios were adjusted for gender; baseline age, body mass index, history of hypertension, history of chronic obstructive pulmonary disease(COPD) and/or pulmonary vascular diseases, history of atrial fibrillation history, systolic blood pressure, estimated glomerular filtration rate (eGFR), high-density lipoprotein cholesterol (HDL-C), sulfonylureas, α-glucosidase inhibitors, thiazolidinediones, dipeptidyl peptidase 4 inhibitors, glucagon-like peptide-1 receptor agonists and sodium-glucose cotransporter-2 inhibitors; and ever use of statin, antiplatelet drugs, diuretics, angiotensin converting enzyme inhibitors/ angiotensin II inhibitors(ACEI/ARBs) , β-receptor blocker and calcium channel blockers(CCBs) during follow-up.

Table S1.

| **Drugs** | **ATC code** |
| --- | --- |
| Antiplatelets | B01AC06, N02BA01, B01AC04, B01AC05, B01AC22, B01AC24, B01AC25, B01AC26, B01AC17, B01AC07 |
| Non-SU insulin secretagogue | A10BX02, 03, 08 |
| DPP4i | A10BH |
| TZD | A10BG |
| α-glucosidase inhibitor | A10BF |
| Sulfonylureas | A10BB |
| Metformin | A10BA02 |
| diuretics | C03 |
| β blocking agents | C07 |
| Calcium channel blockers | C08C, C08D, C08E, C08G |
| GLP-1RA | A10BX04, A10BX07, A10BX10 |
| SGLT2i | A10BX09, A10BX11 |
| Statins | C10AA01-08, C10BA, C10BX01, C10BX03, C10BX05 |
| ACEI | C09A, C09B |
| ARB | C09C, C09D |

Anatomical therapeutic chemical (ATC) codes used to identify pharmaceutical therapies the participants received.

Abbreviations: Non-SU insulin secretagogue: non-sulfonylurea insulin secretagogue; DPP4i: dipeptidyl peptidase 4 inhibitor; TZD: thiazolidinedione; GLP-1RA: glucagon-like peptide-1; SGLT2i: sodium-glucose cotransporter-2 inhibitor; ACEI: angiotensin-converting enzyme inhibitor; ARB: angiotensin II receptor blocker.

Table S2.

| **Outcomes** | **Definition** |
| --- | --- |
| Prior cardiovascular disease (CVD) | Prior CVD was defined as having the any of the following diseases diagnosed prior to baseline: coronary heart disease (CHD, ICD-10 codes I20 – I25), heart failure (HF, ICD-10 code I50), or cerebrovascular disease (ICD-10 codes I60 – I69). |
| Coronary heart disease (CHD) | CHD was defined using the ICD-10 codes of I20 – I25 according to the American Heart Association (AHA).^1^ The event was defined as the first occurrence of the ICD codes above in any follow-up visits after the index day. |
| Hospitalization for heart failure (HF) | Hospitalization for HF was defined as the first occurrence of the diagnosis using the ICD-10 code I50 as the major diagnosis at any hospitalized follow-up visit after the index day. |
| Stroke | Stroke was defined using the ICD-10 codes I60-I64, which included ischemic stroke (I63), intracerebral hemorrhage stroke(I61), subarachnoid hemorrhage stroke(I60), other and unspecified nontraumatic intracranial hemorrhage (I62) and stroke not specified as hemorrhage or infarction(I64). An outcome event of stroke was defined as the first appearance of the diagnosis I60-I64 at any follow-up visit after the index day. |

Definition of the outcome measurements.

Reference:

1. Tsao CW, Aday AW, Almarzooq ZI, et al. Heart Disease and Stroke Statistics-2022 Update: A Report From the American Heart Association. Circulation 2022; 145(8): e153-e639.

Table S3.

|  | **All** | | | | **Propensity score matched** | | | |
| --- | --- | --- | --- | --- | --- | --- | --- | --- |
|  | **Early insulin therapy** | | | **SMD** | **Early insulin therapy** | | | **SMD** |
|  | **No** | **Yes** | |  | **No** | | **Yes** |  |
| N | 3135 | 2289 | |  | 2289 | | 2289 |  |
| Age, mean (SD), y | 57.22 (12.77) | 54.57 (13.27) | | 0.255 | 55.02 (12.62) | | 54.57 (13.27) | 0.034 |
| <40 | 269 (8.58) | 319 (13.9) | |  | 259 (11.3) | | 319 (13.9) |  |
| 40 – 59 | 1536 (49.0) | 1148 (50.2) | |  | 1212 (52.9) | | 1148 (50.2) |  |
| ≥60 | 1330 (42.4) | 822 (35.9) | |  | 818 (35.7) | | 822 (35.9) |  |
| Gender – female, no. (%), | 1326 (42.3) | 850 (37.1) | | 0.106 | 928 (40.5) | | 850 (37.1) | <0.001 |
| Body mass index, mean (SD), kg/m^2^ | 24.71 (10.84) | 24.82 (11.03) | | 0.012 | 24.71 (12.44) | | 24.82 (11.03) | 0.010 |
| <24 | 1517 (48.4) | 1076 (47.0) | |  | 1131 (49.4) | | 1076 (47.0) |  |
| 24 – 27.9 | 1118 (35.7) | 810 (35.4) | |  | 802 (35.0) | | 810 (35.4) |  |
| ≥28 | 500 (15.9) | 403 (17.6) | |  | 356 (15.6) | | 403 (17.6) |  |
| Comorbidities |  |  | |  |  |  | |  |
| Atrial fibrillation | 103 (3.29) | 48 (2.10) | | 0.081 | 58 (2.5) | | 48 (2.1) | 0.029 |
| COPD and/or Pulmonary vascular disease | 99 (3.16) | 31 (1.35) | | 0.101 | 58 (2.5) | | 28 (1.4) | 0.086 |
| Hypertension | 913 (29.1) | 565 (24.7) | | 0.152 | 587 (25.6) | | 565 (24.7) | 0.022 |
| SBP, mean (SD), mmHg | 134.72 (36.62) | 133.82 (46.50) | | 0.024 | 133.85 (40.90) | | 133.82 (46.50) | 0.001 |
| DBP, mean (SD), mmHg | 82.17 (26.60) | 82.37 (28.56) | | 0.016 | 82.43 (26.16) | | 82.37 (28.56) | 0.002 |
| HbA1c, mean (SD), no. (%) | 8.44 (2.89) | 9.73 (3.02) | | 0.451 | 9.16 (3.01) | | 9.73 (3.02) | 0.188 |
| Fasting plasma glucose, median (IQR), mmol/L | 8.44(5.95-13.2) | 9.16(6.09-14.2) | | 0.123 | 8.92 (6.09-14.0) | | 9.16 (6.09-14.2) | 0.034 |
| Serum creatinine, median (IQR), μmol/L | 72.9(60.0-89.0) | 70.0(57.0-89.0) | | 0.007 | 70.7 (58.0-86.0) | | 70.0 (57.0-89.0) | 0.094 |
| eGFR, median (IQR), ml/min/1.73m^2^ | 91.0 (74.1-113) | 97.9 (75.5-125) | | 0.192 | 95.5 (77.7-119) | | 97.9 (75.5-125) | 0.045 |
| Serum total cholesterol, mean (SD), mmol/L | 5.02 (1.58) | 5.03 (1.80) | | 0.012 | 5.10 (1.67) | | 5.03 (1.80) | 0.041 |
| Serum HDL-C, mean (SD), mmol/L | 1.15 (0.35) | 1.08 (0.36) | | 0.157 | 1.14 (0.36) | | 1.08 (0.36) | 0.159 |
| Serum LDL-C, mean (SD), mmol/L | 3.02 (1.14) | 2.89 (1.11) | | 0.098 | 3.08 (1.19) | | 2.89 (1.11) | 0.160 |
| Serum triglyceride, mean (SD), mmol/L | 2.16 (2.78) | 2.42 (3.60) | | 0.078 | 2.29 (3.14) | | 2.42 (3.60) | 0.037 |
| Concomitant antihyperglycemic drugs, no. (%) |  |  |  | |  | |  |  |
| Metformin | 822 (26.2) | 1125 (49.1) | | 0.487 | 644 (28.1) | | 1125 (49.1) | 0.442 |
| Sulfonylureas | 373 (11.9) | 385 (16.8) | | 0.141 | 304 (13.3) | | 385 (16.8) | 0.0099 |
| α-glucosidaseinhibitors | 929 (29.6) | 885 (38.7) | | 0.191 | 737 (32.2) | | 885 (38.7) | 0.135 |
| DPP4is | 109 (3.5) | 272 (11.9) | | 0.320 | 91 (4.0) | | 272 (11.9) | 0.296 |
| GLP-1RAs | 38 (1.2) | 52 (2.3) | | 0.081 | 15 (0.7) | | 77 (3.4) | 0.194 |
| SGLT2is | 101 (3.2) | 161 (7.0) | | 0.173 | 10 (0.4) | | 28 (1.2) | 0.087 |
| TZDs | 125 (4.0) | 76 (3.3) | | 0.036 | 105 (4.6) | | 76 (3.3) | 0.065 |
| Non-SU insulin secretagogues | 373 (11.9) | 302 (13.2) | | 0.039 | 312(13.6) | | 302(13.2) | 0.013 |
| ACEIs/ARBs, no. (%) | 1155 (36.8) | 823 (36.0) | | 0.018 | 763 (33.3) | | 823 (36.0) | 0.055 |
| CCBs, no. (%) | 1173 (37.4) | 820 (35.8) | | 0.033 | 776 (33.9) | | 820 (35.8) | 0.040 |
| β-blockers, no. (%) | 817 (26.1) | 510 (22.3) | | 0.088 | 521 (22.8) | | 510 (22.3) | 0.012 |
| Diuretics, no. (%) | 860 (27.4) | 616 (26.9) | | 0.012 | 548 (23.9) | | 616 (26.9) | 0.068 |
| Statins, no. (%) | 1685 (53.7) | 1212 (52.9) | | 0.016 | 1224 (53.5) | | 1212 (52.9) | 0.011 |
| Antiplatelet drugs, no. (%) | 1271 (40.5) | 819 (35.8) | | 0.098 | 879 (38.4) | | 819 (35.8) | 0.054 |

Baseline characteristics of all the study population, and the propensity score matched population.

Data are presented as number (%), mean (standard deviation) or median (interquartile range).

Abbreviations: SMD, standardized mean difference; SBP, systolic blood pressure; DBP, diastolic blood pressure; HbA1c, hemoglobin A1c; eGFR, estimated glomerular filtration rate; HDL-C, high-density lipoprotein cholesterol; LDL-C, low-density lipoprotein cholesterol; DPP4i, dipeptidyl peptidase 4 inhibitor; GLP-1 RA, glucagon-like peptide-1receptor agonist; SGLT2i, sodium-glucose cotransporter-2 inhibitor; TZD, thiazolidinedione; Non-SU insulin secretagogues, non-sulfonylurea insulin secretagogues; CCBs, calcium channel blockers; ACEI/ARB, angiotensin-converting enzyme inhibitor/angiotensin II receptor blocker.

Table S4.

|  | **Early insulin therapy** | |
| --- | --- | --- |
|  | **No** | **Yes** |
| **No. of patients** | 2289 | 2289 |
| **Outcomes** |  |  |
| **Coronary heart disease** |  |  |
| No of events | 330 | 292 |
| Follow-up time (persons years) | 6314 | 5394 |
| Hazard ratio (95% CI) ^*^ |  |  |
| Model 1 | 1.00 (Reference) | 0.96(0.82 to 1.12) |
| Model 2 | 1.00 (Reference) | 0.98(0.83 to 1.15) |
| Model 3 | 1.00 (Reference) | 1.09 (0.92 to 1.29) |
| Model 4 | 1.00 (Reference) | 1.10 (0.91 to 1.32) |
| **Hospitalization for heart failure** |  |  |
| No of events | 101 | 63 |
| Follow-up time (persons years) | 6886 | 5857 |
| Hazard ratio (95% CI) ^*^ |  |  |
| Model 1 | 1.00 (Reference) | 0.67(0.49 to 0.91) |
| Model 2 | 1.00 (Reference) | 0.65 (0.47 to 0.90) |
| Model 3 | 1.00 (Reference) | 0.65 (0.47 to 0.91) |
| Model 4 | 1.00 (Reference) | 0.69 (0.56 to 0.85) |
| **Stroke** |  |  |
| No of events | 329 | 215 |
| Follow-up time (persons years) | 6544 | 5626 |
| Hazard ratio (95% CI) ^*^ |  |  |
| Model 1 | 1.00 (Reference) | 0.68(0.57 to 0.80) |
| Model 2 | 1.00 (Reference) | 0.67 (0.56 to 0.80) |
| Model 3 | 1.00 (Reference) | 0.70 (0.58 to 0.84) |
| Model 4 | 1.00 (Reference) | 0.64 (0.45 to 0.92) |

Hazard ratios for association between early insulin therapy users versus non-early insulin therapy users and risk of cardiovascular outcomes in the propensity score matched cohort.

*Hazard ratios were estimated using the following models: Model 1, adjusted for gender; baseline age and body mass index; Model 2, Model 1 and further adjusted for baseline history of hypertension, history of chronic obstructive pulmonary disease(COPD) and/or pulmonary vascular diseases, history of atrial fibrillation history, systolic blood pressure, HbA1c, estimated glomerular filtration rate (eGFR), low-density lipoprotein cholesterol(LDL-C) and high-density lipoprotein cholesterol (HDL-C); Model 3, Model 2 and further adjusted for ever use of statin, antiplatelet drugs, diuretics, angiotensin converting enzyme inhibitors/ angiotensin II inhibitors(ACEI/ARBs) , β-receptor blocker and calcium channel blockers(CCBs) during follow-up; and baseline use of metformin, sulfonylureas, α-glucosidase inhibitors, thiazolidinediones, dipeptidyl peptidase 4 inhibitors, glucagon-like peptide-1 receptor agonists and sodium-glucose cotransporter-2 inhibitors.

Table S5.

|  | **Early insulin therapy** | |
| --- | --- | --- |
|  | **No** | **Yes** |
| **No. of patients** | 5429 | 5437 |
| **Outcomes** |  |  |
| **Coronary heart disease** |  |  |
| No of events | 542 | 292 |
| Follow-up time (persons years) | 15310 | 12831 |
| Hazard ratio (95% CI) ^*^ |  |  |
| Model 1 | 1.00 (Reference) | 0.93 (0.80 to 1.08) |
| Model 2 | 1.00 (Reference) | 1.02 (0.88 to 1.19) |
| Model 3 | 1.00 (Reference) | 1.11 (0.95 to 1.30) |
| Model 4 | 1.00 (Reference) | 1.12 (0.94 to 1.33) |
| **Hospitalization for heart failure** |  |  |
| No of events | 167 | 63 |
| Follow-up time (persons years) | 17264 | 13919 |
| Hazard ratio (95% CI) ^*^ |  |  |
| Model 1 | 1.00 (Reference) | 0.68 (0.519 to 0.91) |
| Model 2 | 1.00 (Reference) | 0.70 (0.52 to 0.94) |
| Model 3 | 1.00 (Reference) | 0.69 (0.52 to 0.94) |
| Model 4 | 1.00 (Reference) | 0.67 (0.47 to 0.95) |
| **Stroke** |  |  |
| No of events | 504 | 215 |
| Follow-up time (persons years) | 16124 | 13375 |
| Hazard ratio (95% CI) ^*^ |  |  |
| Model 1 | 1.00 (Reference) | 0.71 (0.60 to 0.83) |
| Model 2 | 1.00 (Reference) | 0.68 (0.58 to 0.81) |
| Model 3 | 1.00 (Reference) | 0.69 (0.58 to 0.82) |
| Model 4 | 1.00 (Reference) | 0.67 (0.54 to 0.82) |

Hazard ratios for association between early insulin therapy users versus non-early insulin therapy users and risk of cardiovascular outcomes weighted with inverse probability treatment weights.

*Hazard ratios were estimated using the following models: Model 1, adjusted for gender; baseline age and body mass index; Model 2, Model 1 and further adjusted for baseline history of hypertension, history of chronic obstructive pulmonary disease(COPD) and/or pulmonary vascular diseases, history of atrial fibrillation history, systolic blood pressure, HbA1c, estimated glomerular filtration rate (eGFR), low-density lipoprotein cholesterol(LDL-C) and high-density lipoprotein cholesterol (HDL-C); Model 3, Model 2 and further adjusted for ever use of statin, antiplatelet drugs, diuretics, angiotensin converting enzyme inhibitors/ angiotensin II inhibitors(ACEI/ARBs) , β-receptor blocker and calcium channel blockers(CCBs) during follow-up; and baseline use of metformin, sulfonylureas, α-glucosidase inhibitors, thiazolidinediones, dipeptidyl peptidase 4 inhibitors, glucagon-like peptide-1 receptor agonists and sodium-glucose cotransporter-2 inhibitors.

Table S6.

|  | **Early insulin therapy** | |
| --- | --- | --- |
|  | **No** | **Yes** |
| **No. of patients** | 2600 | 2000 |
| **Outcomes** |  |  |
| **Coronary heart disease** |  |  |
| No of events | 284 | 151 |
| Follow-up time (persons years) | 8200 | 5062 |
| Hazard ratio (95% CI)^*^ |  |  |
| Model 1 | 1.00 (Reference) | 0.95(0.83 to 1.10) |
| Model 2 | 1.00 (Reference) | 1.03 (0.89 to 1.20) |
| Model 3 | 1.00 (Reference) | 1.11 (0.94 to 1.30) |
| Model 4 | 1.00 (Reference) | 1.30 (1.01 to 1.68) |
| **Hospitalization for heart failure** |  |  |
| No of events | 77 | 24 |
| Follow-up time (persons years) | 8824 | 5238 |
| Hazard ratio (95% CI) ^*^ |  |  |
| Model 1 | 1.00 (Reference) | 0.65(0.47 to 0.90) |
| Model 2 | 1.00 (Reference) | 0.66 (0.47 to 0.93) |
| Model 3 | 1.00 (Reference) | 0.67 (0.49 to 0.91) |
| Model 4 | 1.00 (Reference) | 0.84 (0.48 to 1.48) |
| **Stroke** |  |  |
| No of events | 246 | 99 |
| Follow-up time (persons years) | 8518 | 5150 |
| Hazard ratio (95% CI) ^*^ |  |  |
| Model 1 | 1.00 (Reference) | 0.71(0.59 to 0.85) |
| Model 2 | 1.00 (Reference) | 0.68 (0.56 to 0.81) |
| Model 3 | 1.00 (Reference) | 0.68 (0.56 to 0.83) |
| Model 4 | 1.00 (Reference) | 0.70 (0.52 to 0.93) |

Hazard ratios for association between early insulin therapy users versus non-early insulin therapy users and risk of cardiovascular outcomes, excluding events occurring within 90 days since the index day.

*Hazard ratios were estimated using the following models: Model 1, adjusted for gender; baseline age and body mass index; Model 2, Model 1 and further adjusted for baseline history of hypertension, history of chronic obstructive pulmonary disease(COPD) and/or pulmonary vascular diseases, history of atrial fibrillation history, systolic blood pressure, HbA1c, estimated glomerular filtration rate (eGFR), low-density lipoprotein cholesterol(LDL-C) and high-density lipoprotein cholesterol (HDL-C); Model 3, Model 2 and further adjusted for ever use of statin, antiplatelet drugs, diuretics, angiotensin converting enzyme inhibitors/ angiotensin II inhibitors(ACEI/ARBs) , β-receptor blocker and calcium channel blockers(CCBs) during follow-up; and baseline use of metformin, sulfonylureas, α-glucosidase inhibitors, thiazolidinediones, dipeptidyl peptidase 4 inhibitors, glucagon-like peptide-1 receptor agonists and sodium-glucose cotransporter-2 inhibitors.

Table S7.

|  | **Early insulin therapy** | |
| --- | --- | --- |
|  | **No** | **Yes** |
| **No. of patients** | 2555 | 1975 |
| **Outcomes** |  |  |
| **Coronary heart disease** |  |  |
| No of events | 263 | 139 |
| Follow-up time (persons years) | 8125 | 5036 |
| Hazard ratio (95% CI) ^*^ |  |  |
| Model 1 | 1.00 (Reference) | 1.01 (0.82 to 1.24) |
| Model 2 | 1.00 (Reference) | 1.04 (0.84 to 1.29) |
| Model 3 | 1.00 (Reference) | 1.26 (0.99 to 1.59) |
| Model 4 | 1.00 (Reference) | 1.32 (1.02 to 1.72) |
| **Hospitalization for heart failure** |  |  |
| No of events | 70 | 23 |
| Follow-up time (persons years) | 8713 | 5194 |
| Hazard ratio (95% CI) ^*^ |  |  |
| Model 1 | 1.00 (Reference) | 0.71 (0.55 to 0.91) |
| Model 2 | 1.00 (Reference) | 0.61 (0.37 to 0.99) |
| Model 3 | 1.00 (Reference) | 0.88 (0.52 to 1.52) |
| Model 4 | 1.00 (Reference) | 0.89 (0.50 to 1.59) |
| **Stroke** |  |  |
| No of events | 225 | 90 |
| Follow-up time (persons years) | 8457 | 5115 |
| Hazard ratio (95% CI) ^*^ |  |  |
| Model 1 | 1.00 (Reference) | 0.67 (0.41 to 1.08) |
| Model 2 | 1.00 (Reference) | 0.68 (0.53 to 0.88) |
| Model 3 | 1.00 (Reference) | 0.71 (0.54 to 0.94) |
| Model 4 | 1.00 (Reference) | 0.71 (0.52 to 0.96) |

Hazard ratios for association between early insulin therapy users versus non-early insulin therapy users and risk of cardiovascular outcomes, excluding events occurring within 180 days since the index day.

*Hazard ratios were estimated using the following models: Model 1, adjusted for gender; baseline age and body mass index; Model 2, Model 1 and further adjusted for baseline history of hypertension, history of chronic obstructive pulmonary disease(COPD) and/or pulmonary vascular diseases, history of atrial fibrillation history, systolic blood pressure, HbA1c, estimated glomerular filtration rate (eGFR), low-density lipoprotein cholesterol(LDL-C) and high-density lipoprotein cholesterol (HDL-C); Model 3, Model 2 and further adjusted for ever use of statin, antiplatelet drugs, diuretics, angiotensin converting enzyme inhibitors/ angiotensin II inhibitors(ACEI/ARBs) , β-receptor blocker and calcium channel blockers(CCBs) during follow-up; and baseline use of metformin, sulfonylureas, α-glucosidase inhibitors, thiazolidinediones, dipeptidyl peptidase 4 inhibitors, glucagon-like peptide-1 receptor agonists and sodium-glucose cotransporter-2 inhibitors.

Table S8.

|  | **Early insulin therapy** | |
| --- | --- | --- |
|  | **No** | **Yes** |
| **No. of patients** | 3135 | 2289 |
| **Outcomes** |  |  |
| **Coronary heart disease** |  |  |
| No of events | 542 | 292 |
| Follow-up time (persons years) | 8639 | 5394 |
| Hazard ratio (95% CI) ^*^ |  |  |
| Adjusted for mean HbA1c 6 months after baseline | 1.00 (Reference) | 1.10 (0.93 to 1.31) |
| **Hospitalization for heart failure** |  |  |
| No of events | 167 | 63 |
| Follow-up time (persons years) | 9652 | 5857 |
| Hazard ratio (95% CI) ^*^ |  |  |
| Adjusted for mean HbA1c 6 months after baseline | 1.00 (Reference) | 0.69 (0.55 to 0.86) |
| **Stroke** |  |  |
| No of events | 504 | 215 |
| Follow-up time (persons years) | 9008 | 5626 |
| Hazard ratio (95% CI) ^*^ |  |  |
| Adjusted for mean HbA1c 6 months after baseline | 1.00 (Reference) | 0.81 (0.72 to 0.92) |

Hazard ratios for association between early insulin therapy users versus non-early insulin therapy users and risk of cardiovascular outcomes, adujested for mean glycated hemoglobin a1c level after baseline.

Abbreviation: HbA1c, hemoglobin A1c.*Hazard ratios were estimated adjusted for gender, baseline age, body mass index, baseline history of hypertension, history of chronic obstructive pulmonary disease(COPD) and/or pulmonary vascular diseases, history of atrial fibrillation history, systolic blood pressure, HbA1c, estimated glomerular filtration rate (eGFR), low-density lipoprotein cholesterol(LDL-C) and high-density lipoprotein cholesterol (HDL-C), ever use of statin, antiplatelet drugs, diuretics, angiotensin converting enzyme inhibitors/ angiotensin II inhibitors(ACEI/ARBs) , β-receptor blocker and calcium channel blockers(CCBs) during follow-up, baseline use of metformin, sulfonylureas, α-glucosidase inhibitors, thiazolidinediones, dipeptidyl peptidase 4 inhibitors, glucagon-like peptide-1 receptor agonists, sodium-glucose cotransporter-2 inhibitors, centers where the participants were treated and mean HbA1c 6 months after baseline.

Table S9.

|  | **Early insulin therapy** | |
| --- | --- | --- |
|  | **No** | **Yes** |
| **No. of patients** | 3002 | 2089 |
| **Outcomes** |  |  |
| **Coronary heart disease** |  |  |
| No of events | 538 | 281 |
| Follow-up time (persons years) | 8466 | 4993 |
| Hazard ratio (95% CI) ^*^ |  |  |
| Model 1 | 1.00 (Reference) | 0.93 (0.81 to 1.08) |
| Model 2 | 1.00 (Reference) | 1.00 (0.86 to 1.16) |
| Model 3 | 1.00 (Reference) | 1.06 (0.90 to 1.24) |
| Model 4 | 1.00 (Reference) | 1.08 (0.91 to 1.29) |
| **Hospitalization for heart failure** |  |  |
| No of events | 162 | 59 |
| Follow-up time (persons years) | 9576 | 5431 |
| Hazard ratio (95% CI) ^*^ |  |  |
| Model 1 | 1.00 (Reference) | 0.67 (0.50 to 0.91) |
| Model 2 | 1.00 (Reference) | 0.68 (0.50 to 0.92) |
| Model 3 | 1.00 (Reference) | 0.67 (0.48 to 0.93) |
| Model 4 | 1.00 (Reference) | 0.65 (0.45 to 0.92) |
| **Stroke** |  |  |
| No of events | 496 | 208 |
| Follow-up time (persons years) | 8886 | 5202 |
| Hazard ratio (95% CI) ^*^ |  |  |
| Model 1 | 1.00 (Reference) | 0.72 (0.61 to 0.84) |
| Model 2 | 1.00 (Reference) | 0.69 (0.59 to 0.82) |
| Model 3 | 1.00 (Reference) | 0.68 (0.57 to 0.81) |
| Model 4 | 1.00 (Reference) | 0.67 (0.55 to 0.81) |

Hazard ratios for association between early insulin therapy users versus non-early insulin therapy users and risk of cardiovascular outcomes, excluding participants who had ever used glucagon-like peptide-1 receptor agonists or sodium-glucose cotransporter-2 inhibitors since the index day.

*Hazard ratios were estimated using the following models: Model 1, adjusted for gender; baseline age and body mass index; Model 2, Model 1 and further adjusted for baseline history of hypertension, history of chronic obstructive pulmonary disease(COPD) and/or pulmonary vascular diseases, history of atrial fibrillation history, systolic blood pressure, HbA1c, estimated glomerular filtration rate (eGFR), low-density lipoprotein cholesterol(LDL-C) and high-density lipoprotein cholesterol (HDL-C); Model 3, Model 2 and further adjusted for ever use of statin, antiplatelet drugs, diuretics, angiotensin converting enzyme inhibitors/ angiotensin II inhibitors(ACEI/ARBs) , β-receptor blocker and calcium channel blockers(CCBs) during follow-up; and baseline use of metformin, sulfonylureas, α-glucosidase inhibitors, thiazolidinediones, dipeptidyl peptidase 4 inhibitors.
